# 541 Designing a web-based technology system to support burn disaster response

**DOI:** 10.1093/jbcr/irac012.169

**Published:** 2022-03-23

**Authors:** Kathe M Conlon, Michael A Marano, Robin A Lee, Kevin Montgomery

**Affiliations:** The Burn Center as Saint Barnabas, West orange, New Jersey; The Burn Center at Saint Barnabas, Livingston, New Jersey; The Burn Center at Saint Barnabas, Livingston, New Jersey; IoT/AI, Fremont, California

## Abstract

**Introduction:**

Accurately transmitting information in a burn mass casualty incident (BMCI) is critical. Modern technology, including “apps” and web-based systems coordinated through a central command center addresses this need. The system should 1.) Function as a real-time link between on-scene personnel, local hospitals or trauma centers and regional burn centers; 2.) Organize triage in accordance with accepted burn mass casualty national standards; and 3.) Match acuity of age and percent burn to immediate open beds, either direct from the scene or from secondary hospitals, without overwhelming any one particular facility.

**Methods:**

Extensive literature review was conducted to do a comparative study of similar/existing commercial data management tools; investigate technology designs of mobile apps and web browsers accessible to cloud-based systems, and identify what data and processes needed to be tracked and coordinated across diverse stakeholders to deliver real-time situational awareness for definitive medical planning capabilities.

**Results:**

Development of a Burn Patient Transfer System (BPTS) web-based application with mobile access resulted. The BPTS is series of dashboards designed for specific patient management for both referring (RF) and receiving facilities (RC), coordinated through a central command center. Focus for RFs includes an ability to list number of patients by acuity, addition of new patients, transfer status confirmation and situational awareness throughout the BMCI. RCs report or modify open bed availability of both immediate and eventual beds, for adult or pediatric patients. With this information, appropriate receiving facilities are identified by command personnel to coordinate approval of patient transfer, mode of transport and maintain situational awareness between medical personnel at referring and receiving facilities without exceeding facility surge capability. Clinical experts are responsible for final decisions on a case-by-case basis at both RF and RCs, as defined by a patient transfer algorithm matching patients by age and acuity.

**Conclusions:**

The BPTS is an accessible communications system designed to serve three critical functions; A). Provide a mechanism and platform to report both immediate and surge burn bed capacity; B). Match patient acuity with available open beds at registered medical facilities and burn centers in accordance with ABA Disaster Triage recommendations and C). Track patient movement in real-time. Its core functionality is patient transfer management to and from locations where appropriate care can be delivered based upon clinical needs. This may include initial transfer to a local hospital or trauma center for primary stabilization, or direct to a burn center if and when weather, security and infrastructure permit.

